# Trend analysis and forecast of daily reported incidence of hand, foot and mouth disease in Hubei, China by Prophet model

**DOI:** 10.1038/s41598-021-81100-2

**Published:** 2021-01-14

**Authors:** Cong Xie, Haoyu Wen, Wenwen Yang, Jing Cai, Peng Zhang, Ran Wu, Mingyan Li, Shuqiong Huang

**Affiliations:** 1 Institute of Preventive Medicine Information, Hubei Provincial Center for Disease Control and Prevention, 6 Zhuodaoquan North Road, Wuhan, 430079 Hubei China; 2 Department of Preventive Medicine, School of Health Sciences, Wuhan University, 185 Donghu Road, Wuhan, 430071 China

**Keywords:** Public health, Infectious diseases

## Abstract

Hand, foot, and mouth disease (HFMD) is common among children below 5 years. HFMD has a high incidence in Hubei Province, China. In this study, the Prophet model was used to forecast the incidence of HFMD in comparison with the autoregressive-integrated moving average (ARIMA) model, and HFMD incidence was decomposed into trends, yearly, weekly seasonality and holiday effect. The Prophet model fitted better than the ARIMA model in daily reported incidence of HFMD. The HFMD incidence forecast by the Prophet model showed that two peaks occurred in 2019, with the higher peak in May and the lower peak in December. Periodically changing patterns of HFMD incidence were observed after decomposing the time-series into its major components. In specific, multi-year variability of HFMD incidence was found, and the slow-down increasing point of HFMD incidence was identified. Relatively high HFMD incidences appeared in May and on Mondays. The effect of Spring Festival on HFMD incidence was much stronger than that of other holidays. This study showed the potential of the Prophet model to detect seasonality in HFMD incidence. Our next goal is to incorporate climate variables into the Prophet model to produce an accurate forecast of HFMD incidence.

## Introduction

Hand, foot, and mouth disease (HFMD) is common among children below 5 years. It is mainly caused by various enteroviruses including enterovirus 71 (EV71) and Coxsackie virus A16 (CVA16) and occurs mostly in spring to autumn. HFMD is transmitted through close personal contact, exposure to feces, contaminated objects, and surfaces of an infected person^1–4^.

HFMD presents a growing public concern with considerable health and economic effect in affected areas because of its high incidence, seasonal pattern and frequent outbreaks. HFMD was estimated to cause 96,900 age-standardised disability adjusted life years per annum in eight high-burden countries in East and Southeast Asia^5^. In China, more than 7.2 million cases of HFMD were reported to the national enhanced surveillance system during 2008–2012^6^, with the highest yearly incidence (114.48 per 100,000) among the infectious diseases in 2004–2013^7^. In Hubei Province, China, HFMD has the highest incidence among legally reported infectious diseases, with an average annual incidence rate of 123.1 per 10,000 from 2009 to 2015^8^.

Given the heavy disease burden of HFMD among children in Hubei Province, China, the trend and seasonal variation of HFMD incidence need to be explored and forecast. Many classic models, including the Autoregressive-integrated Moving Average (ARIMA) model, Artificial Neural Networks, Support Vector Regression and Long Short-Term Memory, were used to analyse and forecast the incidence of HFMD^9–12^.

However, all above models require professional analysts and perform poorly when processing daily data, which complicate the analysis of the weekly seasonality of time series data. All these disadvantages are determined as motivation for the Prophet model. Adopting a generalized additive model (GAM) formulation, the Prophet model can model multiple periods of seasonality simultaneously. It is fast in its fitting procedure and robust to large outliers, missing values, and dramatic changes^13–15^. To date, the Prophet model has been used in many subjects including infectious diseases such as flu^16^ and COVID-19^17,18^. However, studies on using the Prophet model to analyse and forecast HFMD incidence are lacking.

Therefore, motivated by the merits of the Prophet model and the urgent need to explore the trends of HFMD, this study aims to forecast the incidence of HFMD and explore the trends, seasonality and holiday effect on HFMD incidence by using the Prophet model, which will be of great help in initiating guidance planning and effective intervention measures for HFMD-prevention. Meanwhile, this study compared the simulating and forecasting abilities of the Prophet model with the ARIMA model, to evaluate the accuracy of the Prophet model in forecasting the daily incidence of HFMD.

## Materials and methods

### Materials

#### Data sources

Since May 2008, the Chinese Ministry of Health has categorised HFMD as a class C communicable disease, which means HFMD incidence must be reported to the National Communicable Disease Surveillance Network within 24 h after diagnosis^19^. Unlike other sentinel surveillance data, the data collected from this network are population wide, which can be more representative for the actual HFMD epidemic. HFMD was reported voluntarily before May 2008; thus, the data were sparse before. Although the Prophet model is robust to missing values, data from 2009 to 2018 were used for analysis to obtain accurate forecast.

In this study, the daily incidence of HFMD data in Hubei Province were collected from the National Communicable Disease Surveillance Network with patients’ address in Hubei Province, including confirmed cases and clinically diagnosed cases. The date of onset was used to establish the occurrence of cases in this study.

#### Processing

To obtain accurate forecasting models and avoid over-fitting, we used the HFMD incidence data from 2009 to 2017 as the training set and the data in 2018 as the test set. The first step in implementing an ARIMA model is to determine whether or not the time series data are stationary. Due to the significant seasonality, HFMD incidence data were non-stationary. Thus, we used log-transform and differencing in implementing the ARIMA and Prophet models. We divided the daily records into four seasons (i.e. spring: March, April, May; summer: June, July, August; fall: September, October, November; and winter: December, January, February).

### Methods

#### ARIMA model

The ARIMA is a generalised model of Autoregressive Moving Average (ARMA) that combines Autoregressive (AR) process and Moving Average (MA) processes and builds a composite model of the time series^20^. As acronym indicates, (*p*, *d*, *q*) captures the key elements of the model:

- *p* = number of autoregressive terms;
- *d* = number of non-seasonal differences;
- *q* = number of moving-average terms.

With seasonal time series data, the Seasonal-ARIMA (SARIMA) model, which incorporates non-seasonal and seasonal factors in a multiplicative model, need to be estimated. The general form of SARIMA model is denoted as *ARIMA* (*p*, *d*, *q*) × (*P*, *D*, *Q*) *S*, which can be described with the following parameters:

- *P* = number of seasonal autoregressive terms;
- *D* = number of seasonal differences;
- *Q* = number of seasonal moving-average terms;
- *S* = the periodic terms.

Considering that the reported incidence of the HFMD dataset presents strong seasonal tendencies, the adoption of SARIMA is a good choice. The SARIMA implemented here was embedded with a periodicity of 365 days (S = 365).

#### Prophet model

The Prophet model was designed for the analysis and forecast of time-series data introduced by Taylor and Letham and available in Python and R^14^. It adopts a Bayesian-based curve fitting method to smooth and forecast time-series data. The formulation of the Prophet model is similar to a GAM, including trend, seasonality and holidays:

1 $$ {\text{y}}(t) = g(t) + s(t) + h(t) + \varepsilon_{t} , $$

where g(t) is the trend function, which represents non-periodic changes in time series values, and s(t) represents periodic changes (e.g. weekly and yearly seasonality). Many studies have reported that HFMD has obvious yearly seasonality, but few studies have focused on weekly seasonality. The present study aims to fill this research. h(t) represents the effects of holidays. In this study, seven holidays were considered, including New Year, Spring Festival, Tomb-sweeping Festival, May Day, Dragon Boat Festival, Mid-autumn Festival and National Day (The dates of the above festivals in 2009–2018 are shown in Supplementary Table S2 online). ε_t_ is the error term and assumes to be normally distributed in this study. This GAM specification of the Prophet model allows analysts to explore different model specifications interactively and flexibly.

The Prophet model works best with time series that have strong seasonal effects and several seasons of historical data. Compared with traditional exponential smoothing models, the Prophet model can extract trends and periodic signals with different time scales more easily with multiple periods and has no requirements regarding the regularity of measurement spacing. The Prophet model facilitates the analysis of different time-series and filters out noise and outliers from datasets^13^. These features make it attractive for the analysis of HFMD incidence. All scripts have been written in R statistical programming language.

#### Assessment metric

We have used different metrics for both models and these metrics are listed below:

- Root-mean-square error (RMSE), $RMSE = \sqrt {\frac{1}{n}\sum\nolimits_{i = 1}^{n} {(a - c)^{2} } }$, where $n$ is the number of samples, $a$ is the actual value; and $c$ is the predicated value. RMSE measures the differences or residuals between actual and predicated values.
- Mean-absolute-error (MAE), $MAE = \frac{{|a_{1} - c_{1} | + |a_{2} - c_{2} | + \cdots + |a_{n} - c_{n} |}}{n}$.
- Mean-absolute-percentage-error (MAPE), $MAPE = \frac{1}{n}\sum\nolimits_{i = 1}^{n} {\frac{{|a_{i} - c_{i} |}}{{a_{i} }}}$.
- Absolute-error (AE), $AE = \frac{{|a_{1} - c_{1} | + |a_{2} - c_{2} | + \cdots + |a_{n} - c_{n} |}}{{|a_{1} - \overline{a}| + |a_{2} - \overline{a}| + \cdots + |a_{n} - \overline{a}|}}$, where $\overline{a}$ is the mean of actual value.
- Pearson correlation coefficient (Pearson’s *r*), Pearson’s *r* = $\frac{{n\sum a_{i} c_{i} - \sum a_{i} \sum c_{i} }}{{\sqrt {n\sum a_{i}^{2} - \left( {\sum a_{i} } \right)^{2} } \sqrt {n\sum c_{i}^{2} - \left( {\sum c_{i} } \right)^{2} } }}$.

We used the RMSE, MAE, MAPE, AE, Pearson’s *r* and the fitting effect diagram to measure the performance of the models. Lower values indicate a better fit of the data. The best fitted model will be used to forecast the daily reported incidences of HFMD in 2019.

### Ethics approval

The data of this study is from the National Communicable Disease Surveillance Network which is open to specific users with some permission. The data processing used secondary statistical table instead of private case information, so the anonymity of the data was maintained. In all, ethical approval is not required for this study.

## Results

### General information

**Figure 1:**
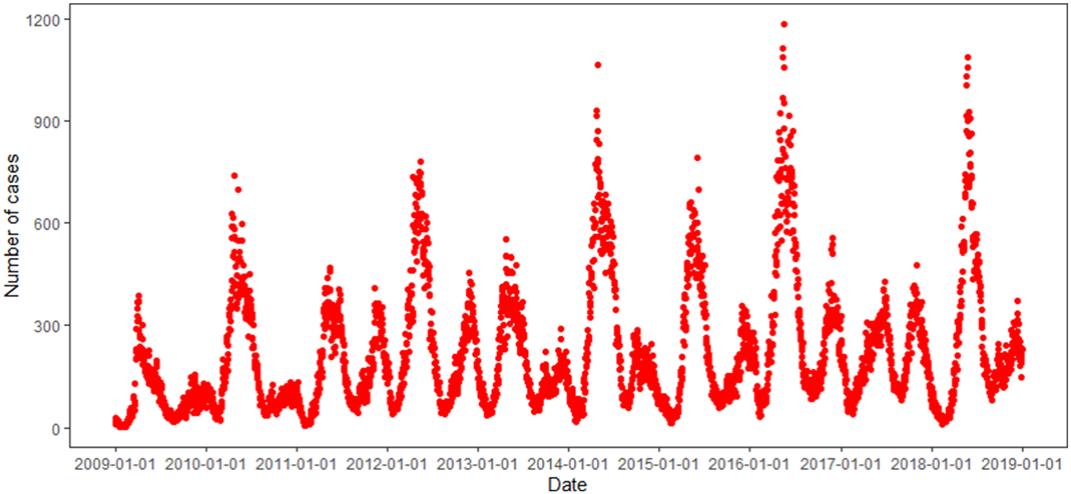
The temporal distribution of daily reported HFMD incidence from 2009 to 2018 in Hubei Province, China.

A total of 784,618 cases were reported, increasing from 33,211 cases in 2009 to 92,376 cases in 2018, with an annual increase of 11.11% during this period. Figure 1 illustrates an obvious seasonal pattern and upward trend in the reported incidence of HFMD from 2009 to 2018 in Hubei Province. The reported incidence of HFMD showed two peaks in a year with the higher peak observed in April–June and the lower peak in September–October. Furthermore, an epidemic peak appeared every other year from 2009 to 2018, which indicated that the peak of the reported incidence in even years was higher than that in odd years.

**Figure 2:**
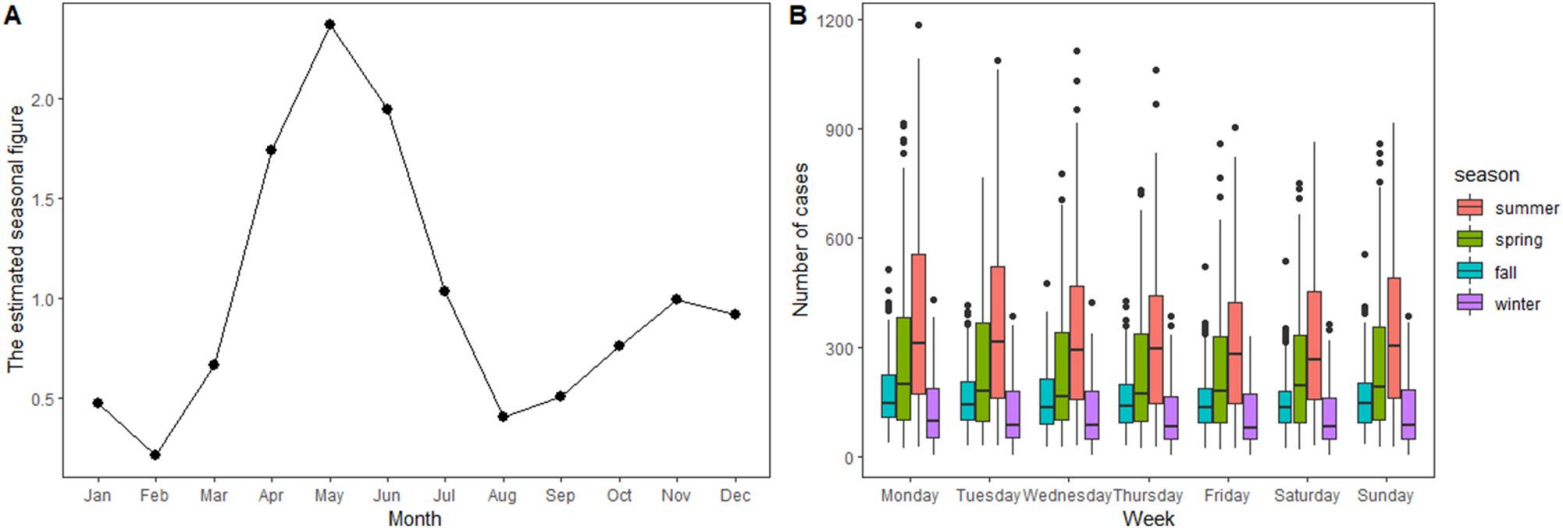
The decomposition using moving averages (**A**), and the boxplot of the daily reported incidence of HFMD for the same days of the week in four seasons in Hubei Province, China by R4.0.3 software (https://www.R-project.org) (**B**).

The seasonal pattern was supported by the moving averages decomposition approach (Fig. 2A), in which the seasonal effect is the seasonal average divided by the average of each day. Clear seasonal peaks and an evident 12-month cyclical process occurred. Figure 2B shows that the daily reported incidence of HFMD was the highest in summer, followed by spring while lower in fall and winter. Meanwhile, the reported incidence of HFMD was higher on Monday and Sunday and lower on Saturday and Friday in each of the seasons, except spring (lower incidence on Wednesday and Thursday). This preliminary assessment revealed the possible day-of-week and seasonal effects in the daily reported incidence of HFMD in Hubei Province. Detailed and reliable analyses are warranted using a statistical time-series analysis.

### Forecast of the ARIMA and Prophet models

The ARIMA and Prophet models were used based on the dataset of reported HFMD incidence. As mentioned before, the training set is the dataset from 2009 to 2017, and the test set is the dataset in 2018.

For ARIMA, the series showed a non-stationary mean (Fig. 1). Thus, we stabilised the daily reported incidences of HFMD by taking first difference. Then, the observations were examined using the augmented Dickey–Fuller (ADF) test (ADF =  − 11.79, *P* < 0.01), which was indicative of an obviously stationary incidence series. All further statistical procedures were performed on the log-transformed HFMD incidence. Basing from the distribution characteristics and autocorrelation graph and partial autocorrelation graph, we conducted several possible ARIMA models for the daily reported incidence of HFMD. We set the Akaike Information Criterion (AIC), and lower AIC values indicate better fit model for the data. Among several possible ARIMA models, ARIMA (4,1,4) (0,1,0) [365] and ARIMA (5,1,3) (0,1,0) [365] hold lower AIC values; thus, both models were selected for the following comparison. The Ljung–Box test was used to measure the autocorrelation of the residual of ARIMA forecast across a specified number of time lags. Although the AIC value of ARIMA (4,1,4) (0,1,0) [365] was smaller than that of ARIMA (5,1,3) (0,1,0) [365], the forecast of ARIMA (4,1,4) (0,1,0) [365] failed the Ljung–Box test. Thus, the model of ARIMA (5,1,3) (0,1,0) [365] was considered to be best fit for the data (details in Supplementary Table S1 online).

For the Prophet model, it is easy and friendly to forecast at daily reported HFMD of its piecewise trends, diverse seasonality, and changing holidays. We implemented the trend model with a saturating growth, and the carrying capacity of the logistic growth model was set as 8.5. The change-points were automatically selected and the number of change-points was set as 25. We set the interval width as 0.95 and the number of uncertainty samples as 1000.

To determine which model is the best fit for the data within Prophet and ARIMA(5,1,3) (0,1,0) [365], we set the criteria as follows (depicted in Table 1): RMSE, MAE, MAPE, AE and Pearson's *r* values were calculated in the training and test processing for the two models. Table 1 shows that Prophet and ARIMA fitted very well in the training sets and that ARIMA was slightly better than Prophet. However, in the test sets, ARIMA was much worse fitting than Prophet.

**Table 1:** RMSE, MAE, MAPE, AE and Pearson's *r* values calculated in the training and test phases for the two models.

Model	ARIMA (5,1,3) (0,1,0) [365]	Prophet
**Training set**		
RMSE	50.70	94.66
MAE	31.85	61.99
MAPE	0.17	0.34
AE	0.24	0.46
Pearson's *r*	0.96	0.84
**Test set**		
RMSE	264.28	126.17
MAE	171.09	87.64
MAPE	0.56	0.69
AE	1.00	0.51
Pearson's *r*	0.54	0.84

**Figure 3:**
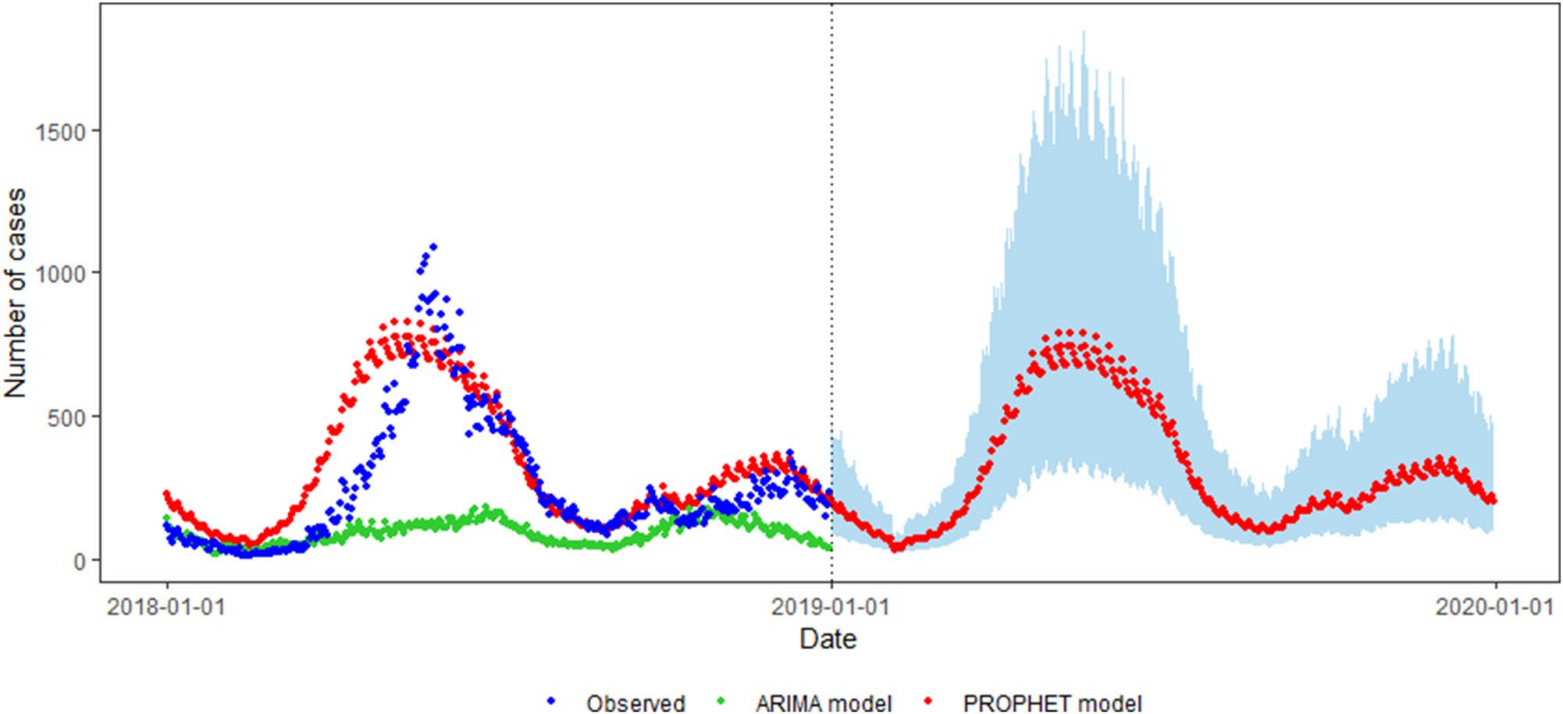
Visualization of fitting for both models in 2018 and forecast of HFMD using the Prophet model in 2019.

Figure 3 visualises the goodness of fitting for both models and supports the results reported on Table 1. In the test set, the ARIMA model was over-fitting with poor generalisation ability, whereas the Prophet model was fitted well and captured the seasonality. Basing from the forecast on the test set, we concluded that the forecasting of Prophet model was better than that of ARIMA in the daily reported incidence of HFMD in this study.

Given its well-fitting, the Prophet model was used to forecast the daily reported incidence of HFMD in 2019. The Prophet model forecast two peak, with the higher peak of 790 in May and the lower peak of 349 in December. The forecasting higher peak in 2019 was slightly lower than the highest peak in 2018, which was in line with the epidemic mentioned above.

### Decomposed components of Prophet

**Figure 4:**
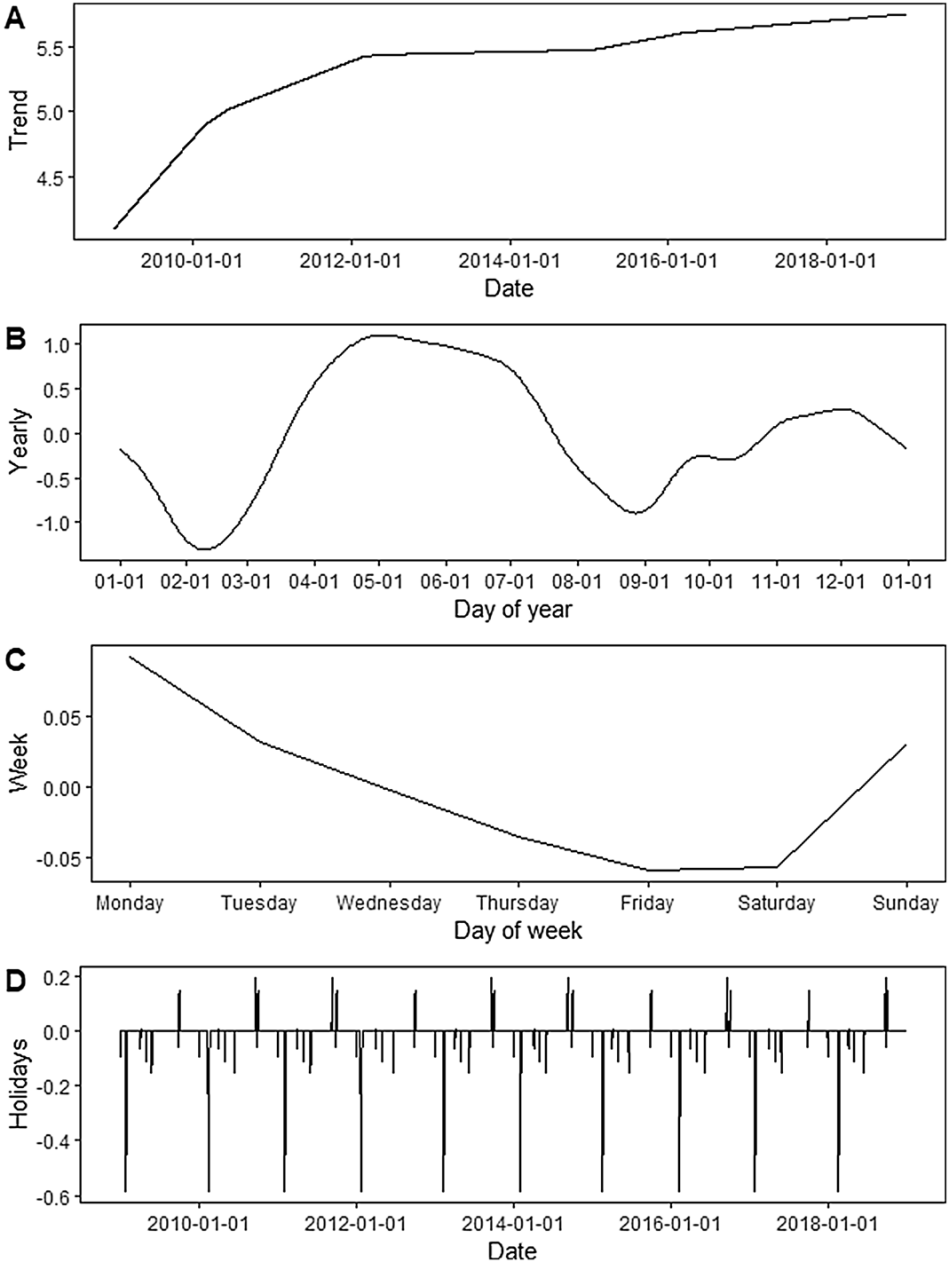
The decomposed components of the daily reported cases of HFMD time series in Hubei Province, China, including the effect of trend (**A**), the yearly (**B**), the weekly (**C**), and the holidays (**D**).

The estimated reported incidence of HFMD is a combination of trend effect, yearly seasonality effect, weekly seasonality effect and holiday effect. These components are shown by Fig. 4, from which an apparently increasing trend in the reported incidence of HFMD can be observed from 2009 to 2018. The trend effect increased rapidly before 2012 and presented a slower increase after 2012. For yearly seasonality, two apparent local maxima appeared in early May and early December and two apparent local minima appeared in mid-February and later August. The day-of-week curve exhibited that the reported incidence of HFMD sharply fell from Monday to Friday, reached the nadir on Friday and then steadily increased from Friday to next Monday. The holiday component indicates that the holidays around the New day, Spring Festival, Tomb-sweeping Festival, May day and Dragon Boat Festival exerted positive effects, but Mid-autumn Festival and National Day exerted negative effects. The effect of Spring Festival was much greater than those of the other holidays.

## Discussion

Many studies have proven that early forecast of HFMD epidemics facilitates effective prevention strategies, and the ARIMA model is commonly used to forecast HFMD epidemics^9,11^. A study by the Chinese Center for Disease Control and Prevention on HFMD showed that each extra day increase in onset-to-diagnosis interval is associated with a 1% higher risk of HFMD mortality^6^. Therefore, forecasting the incidence of HFMD at the daily scale can help public health professionals implement prevention and control measures in a timely and accurate manner, thereby reducing the risk of HFMD mortality. However, most studies of forecasting HFMD epidemics based on the ARIMA model used weekly, monthly and yearly data but rarely daily data^11,21,22^. The ARIMA model is easy to over-fit with poor generalisation ability when processing daily data. However, the Prophet model has strong generalisation ability and performs better in the forecast based on daily data.

Recently, neural networks (including back propagation neural network, long short-term memory neural networks and convolutional-recurrent neural network, etc.) have been used to forecast HFMD incidence on the basis of monthly data^10,12,23^. Comparison of the fitting indexes (RMSE and MAPE) of the neural network for HFMD forecast in a previous study with the Prophet model in this study shows that the Prophet model performed much better. This result is not only due to Prophet’s good forecasting ability, but also to the rich information provided by daily data. In addition, this GAM specification in the Prophet model could help user decompose time serious into trends, yearly, weekly seasonality and holidays by the nature of the time series.

The continuously increasing trends of HFMD may contribute to the periodic rotation and dominance of the three pathogens, EV71, CVA16 and CVA6, which appeared to evade from large-scale population immunity. It also contributed to some events, which may affect the epidemics of HFMD^24^. Since the initiation of surveillance in 2008, the reported incidence of HFMD has increased sharply because of improvements in reporting and surveillance of HFMD^6^. In 2010, the China Health Commission issued the ‘Chinese guidelines for the diagnosis and treatment of hand, foot and mouth disease (2010 edition)’ to help the health sectors and residents to prevent and deal with HFMD. With the increasing awareness of HFMD, relevant prevention and control measures have increasingly been strengthened. As a result, the rising trend of HFMD slowed down since 2010. In 2016, three inactivated monovalent EV71 vaccines were licensed in China, which consequently decreased the proportion of EV71 associated HFMD^25^. In 2018, the Health Commission of Hubei provincial issued a document on HFMD, including a series of prevention and control measures, such as strengthening surveillance, increasing EV71 vaccination and improving diagnostic ability, which exerted positive effects on reducing the incidence of HFMD^26^. Effective vaccines against EV71 have been prepared in China but are only available in the private market and require out of-pocket payment. For other countries, paediatric vaccination programs have not been introduced^27^. Moreover, the vaccine against EV71 lacks cross-protection against other HFMD viruses such as CVA16.

The yearly seasonality of HFMD incidence has always been the focus of HFMD-related researchers. The Chinese Center for Disease Control and Prevention showed that the incidence of HFMD in China held different seasonal variation in the south and north: it has a summer peak in northern China and a spring and autumn peak in southern China^6^, which is consistent with the results of this study. Previous studies suggested that climate (including temperature, humidity, precipitation, air pressure, etc.) is related to the yearly seasonality of HFMD incidence. Our next goal is to incorporate climate variables into the Prophet model to produce a more accurate forecast of HFMD incidence, with a view to help establish an effective HFMD outbreak warning system.

Compared with the trends, yearly seasonality and holidays effect, the impact of weekly seasonality on HFMD incidence was relatively weak. HFMD in Hubei Province was common on people aged 6 months and 5 years old^8^, and their average incubation period was estimated to be 4.4 days^28^. This result meant that the number of HFMD cases today may be affected by various factors 4 days ago. For people aged 6 months to 5 years old, their caregivers are an important factor affecting HFMD^29^. However, the trajectory of children’s caregivers was changeable and hard to track every week. In the future, the reasons behind the weekly seasonality of HFMD incidence need to be further explored.

Holidays mean that children do not attend school, which may have a positive effect in reducing the transmission of HFMD via schools^30^. However, holidays increased the contact between children and their family members, which may increase the transmission of HFMD via families. Children go to public places during school holidays, which is a risk factor of HFMD transmission^31^. A previous study indicated that school holidays have limited effects on HFMD transmission which may be partially explained by relatively short school holiday^32^. Another study showed that school closure during public holidays exerts a relatively small effect of mitigating transmission, and the effect cannot be solely attributable to public holidays because of seasonal effect^30^. The holidays included in this study were relatively short, lasting 3 or 7 days and the incidence of HFMD in Hubei Province showed obvious seasonality. Therefore, the specific mechanism underlying positive or negative holiday effects are difficult to elucidate. In-depth analysis on specific mechanism of holidays is urgently needed. However, the effects of Spring Festival were much greater than those of other holidays, which can be attributed to the fact that Spring Festival occurred during the winter vacation for a month. Some studies showed that summer or spring school vacations with duration longer than a month are associated with low HFMD transmission^33^, which could explain the relatively obvious negative effect of Spring Festival.

Compared with the ARIMA model, the Prophet model can provide a more accurate forecast of HFMD incidence at a tinier scale. This study showed the potential of the Prophet model to detect seasonality in HFMD incidence. Our next goal is to incorporate climate variables into the Prophet model to produce an accurate forecast of HFMD incidence and help establish an effective HFMD outbreak warning system.

## Supplementary Information

Supplementary Tables.
