# Supplementary material for: Trend analysis and forecast of daily reported incidence of hand, foot and mouth disease in Hubei, China by Prophet model
Source: Sci Rep. 2021 Jan 14;11:1445. doi: 10.1038/s41598-021-81100-2 (PMC7809027; doi:10.1038/s41598-021-81100-2)
Supplement: Supplementary file 1 — Supplementary Tables. [file 41598_2021_81100_MOESM1_ESM.docx]

Trend analysis and forecast of daily reported incidence of hand, foot and mouth disease in Hubei, China by Prophet model

Cong Xie^1, #^, Haoyu Wen^2, #^, Wenwen Yang^1^, Jing Cai^1^, Peng Zhang^1^, Ran Wu^1^, Mingyan Li^1*^, Shuqiong Huang^1*^

1 Institute of Preventive Medicine Information, Hubei Provincial Center for Disease Control and Prevention, 6 Zhuodaoquan North Road, Wuhan, Hubei 430079, China

2 Department of Preventive Medicine, School of Health Sciences, Wuhan University, 185 Donghu Road, Wuhan 430071, China

# These authors contributed equally to this work.

*** Correspondence:**Mingyan Li
limingyan@163.com

&

Shuqiong Huang
hsq0817@outlook.com

Supplementary Table S1: The AIC and Ljung-Box Q statistic for several possible ARIMA models

| ARIMA model | AIC | Ljung-Box Q statistic | | | |
| --- | --- | --- | --- | --- | --- |
|  |  | df | *P* value | df | *P* value |
| (5,1,1)(0,1,0)[365] | 33.39 | 6 | 0.483 | 12 | 0.000 |
| (4,1,1)(0,1,0)[365] | 34.39 | 6 | 0.741 | 12 | 0.000 |
| (3,1,1)(0,1,0)[365] | 36.44 | 6 | 0.772 | 12 | 0.000 |
| (4,1,2)(0,1,0)[365] | 34.41 | 6 | 0.326 | 12 | 0.000 |
| (3,1,2)(0,1,0)[365] | 37.58 | 6 | 0.731 | 12 | 0.000 |
| (5,1,2)(0,1,0)[365] | 24.13 | 6 | 0.002 | 12 | 0.002 |
| (5,1,3)(0,1,0)[365] | 11.34 | 6 | 0.361 | 12 | 0.090 |
| (4,1,3)(0,1,0)[365] | 38.35 | 6 | 0.577 | 12 | 0.000 |
| (4,1,4)(0,1,0)[365] | -28.94 | 6 | 0.000 | 12 | 0.001 |

Supplementary Table S2: The date of the holiday item of the Prophet model

| year | New day | Spring Festival | Tomb-sweeping Festival | May day | Dragon Boat Festival | Mid-autumn Festival | National Day |
| --- | --- | --- | --- | --- | --- | --- | --- |
| 2018 | 12.30-1.1 | 2.15~2.21 | 4.5-4.7 | 4.29-5.1 | 6.16-6.18 | 9.22-9.24 | 10.1-10.7 |
| 2017 | 12.31-1.2 | 1.27-2.2 | 4.2-4.4 | 4.29-5.1 | 5.28-5.30 | 10.4 | 10.1-10.8 |
| 2016 | 1.1-1.3 | 2.7-2.13 | 4.2-4.4 | 4.30-5.2 | 6.9-6.11 | 9.15-9.17 | 10.1-10.7 |
| 2015 | 1.1-1.3 | 2.18-2.24 | 4.4-4.6 | 5.1-5.3 | 6.20-6.22 | 9.27 | 10.1-10.7 |
| 2014 | 1.1-1.3 | 1.31-2.6 | 4.5-4.7 | 5.1-5.3 | 6.2-6.4 | 9.8-9.10 | 10.1-10.7 |
| 2013 | 1.1-1.3 | 2.9-2.15 | 4.4-4.6 | 4.29-5.1 | 6.10-6.12 | 9.19-9.21 | 10.1-10.7 |
| 2012 | 1.1-1.3 | 1.22-2.28 | 4.2-4.4 | 4.29-5.1 | 6.22-24 | 9.30 | 9.30-10.7 |
| 2011 | 1.1-1.3 | 2.2-2.8 | 4.3-4.5 | 4.30-5.2 | 6.4-6.6 | 9.10-9.12 | 10.1-10.7 |
| 2010 | 1.1-1.3 | 2.13-2.19 | 4.3-4.5 | 5.1-5.3 | 6.14-6.16 | 9.22-24 | 10.1-10.7 |
| 2009 | 1.1-1.3 | 1.25-1.31 | 4.4-4.6 | 5.1-5.3 | 5.28-5.30 | 10.5 | 10.1-10.8 |
